# WormBase: a modern Model Organism Information Resource

**DOI:** 10.1093/nar/gkz920

**Published:** 2019-10-23

**Authors:** Todd W Harris, Valerio Arnaboldi, Scott Cain, Juancarlos Chan, Wen J Chen, Jaehyoung Cho, Paul Davis, Sibyl Gao, Christian A Grove, Ranjana Kishore, Raymond Y N Lee, Hans-Michael Muller, Cecilia Nakamura, Paulo Nuin, Michael Paulini, Daniela Raciti, Faye H Rodgers, Matthew Russell, Gary Schindelman, Kimberly V Auken, Qinghua Wang, Gary Williams, Adam J Wright, Karen Yook, Kevin L Howe, Tim Schedl, Lincoln Stein, Paul W Sternberg

**Affiliations:** 1 Informatics and Bio-computing Platform, Ontario Institute for Cancer Research, Toronto, ON M5G0A3, Canada; 2 Division of Biology and Biological Engineering 156–29, California Institute of Technology, Pasadena, CA 91125, USA; 3 European Molecular Biology Laboratory, European Bioinformatics Institute, Wellcome Trust Genome Campus, Hinxton, Cambridge CB10 1SD, UK; 4 Wellcome Trust Sanger Institute, Wellcome Trust Genome Campus, Hinxton, Cambridge CB10 1SA, UK; 5 Department of Genetics, Washington University School of Medicine, St Louis, MO 63110, USA

## Abstract

WormBase (https://wormbase.org/) is a mature Model Organism Information Resource supporting researchers using the nematode *Caenorhabditis elegans* as a model system for studies across a broad range of basic biological processes. Toward this mission, WormBase efforts are arranged in three primary facets: curation, user interface and architecture. In this update, we describe progress in each of these three areas. In particular, we discuss the status of literature curation and recently added data, detail new features of the web interface and options for users wishing to conduct data mining workflows, and discuss our efforts to build a robust and scalable architecture by leveraging commercial cloud offerings. We conclude with a description of WormBase's role as a founding member of the nascent Alliance of Genome Resources.

## INTRODUCTION

WormBase (https://wormbase.org/) provides researchers using *Caenorhabditis elegans* as a model system a carefully curated and highly integrated view of decades of experimental data (1,2). With deep roots as an electronic resource pre-dating the rise of the web and driven by a scientific community well known for open sharing, WormBase has grown from supporting ∼300 active labs to over 1400 today. WormBase now spans multiple nematode species and annotations across the range of modern biology and a wealth of tools to assist in experimental design. In addition, WormBase has incorporated many datasets unique to the system, such as the *C. elegans* developmental lineage and neuronal connectivity. WormBase serves a global community of researchers and is an essential part of daily ‘worm work’.

Backed by a team of biological curators, WormBase extracts experimental observations from the literature placing them in a searchable, highly cross-referenced and computable environment. To accelerate the rate and coverage of curation, WormBase solicits direct contributions from domain experts via community curation interfaces and relies on automated methods when feasible. In this update, we describe our progress on curating the primary scientific literature; introduce new interfaces for querying and visualizing sequence and phenotype ontologies; and discuss improvements to the genome browser. We conclude with a discussion of data mining services at WormBase and introduce the cloud-native architecture that drives the WormBase website.

WormBase follows a 2-month release schedule and is a fully open source and open access resource available under a Creative Commons Public Domain license. We provide a large variety of precomputed data files in a variety of useful formats on our FTP site (ftp://ftp.wormbase.org).

Services like WormBase have long been known as Model Organism Databases, or MODs. But as the work that WormBase performs greatly extends beyond the creation of a database for a model organism, and as the Alliance of Genome Resources increases its footprint, we propose a new, more expansive moniker for such projects: MOIRs—Model Organism Information Resources.

## CURATION

### New ‘Author First Pass’ pipeline

Since 2009, WormBase has engaged authors, via an Author First Pass (AFP) pipeline (3), to help classify data types and entities in their recently published papers. Over the past year, we greatly improved the existing pipeline by combining Text Mining (TM) approaches and AFP into a single application to enhance community curation. Specifically, we use named entity recognition and document classification methods (e.g. Support Vector Machines; (4)) to extract biological entities from the full text of articles and flag data types, respectively, and present the results of these methods to authors in a pre-populated web-based form. The entities extracted are alleles, genes, species, strains and transgenes; and the datatypes flagged include gene expression data, genetic, physical, and regulatory interaction data, and phenotypic data. Additional data types routinely curated for inclusion in WormBase but not currently flagged by our pipeline can still be reported by authors.

As before, corresponding authors are contacted via email shortly after their publication is incorporated into the WormBase curation database. The email message contains a link to a form where authors simply need to validate the results of machine-based pipelines, rather than enter all information *denovo*, or flag additional data types. Wherever possible, we are also providing links from the new AFP interface to structured data submission forms, e.g. phenotypes and expression patterns, giving authors the opportunity to contribute more detailed curation that can be incorporated into WB with minimal curator review. By leveraging TM techniques in our new AFP pipeline, we hope to lessen the participatory burden for authors, while at the same time receive valuable feedback on the performance of our TM tools.

### Community curation of phenotypes

WormBase is committed to curating all phenotypes reported in the *C*. *elegans* and nematode literature. However, after 15 years of backlog and contemporaneous curation, the reporting rate of new phenotypes now outpaces the ability for WormBase curators to manually curate phenotypes and stay up-to-date with the literature. For example, WormBase phenotype curators (generally 1–2 FTE) can typically curate 200–300 phenotype papers per year, but now more than 700 phenotype papers are being published each year. Since October 2015, to achieve better coverage of reported phenotypes, WormBase has been soliciting direct contributions of phenotypes by sending emails to authors of papers predicted to contain phenotype information requesting that they submit phenotype data via our phenotype data submission form (https://wormbase.org/submissions/phenotype.cgi) or via our WormBase Phenotype Worksheet. In June 2018, we initiated a large-scale outreach approach whereby we email all first authors of these papers (or corresponding authors if first author contact information is unavailable) in one large batch using automated approaches to expedite the email delivery process, and repeat this process every 3 months.

Since then, WormBase has sent out 8144 emails to authors requesting phenotype data (sometimes emailing about the same paper another time after a 6-month waiting period) and has received feedback (curation or flags for no phenotype data) for 476 papers of 4748 papers for which we sent a request (∼10% response rate). From June 2018 to August 2019, WormBase received 3227 phenotype annotations for 440 papers made by 324 distinct community curators, greatly improving our coverage of phenotypes reported in the literature. From the inception of the community phenotype curation pipeline in October 2015 to August 2019, WormBase has received a total of 7118 phenotype annotations for 978 papers made by 610 distinct community curators. WormBase greatly appreciates this community effort and would like to thank the *C. elegans* research community for their dedication and effort in maintaining phenotype data at WormBase.

### Protein–protein interaction curation current to-date

WormBase now houses the most up-to-date and complete protein–protein interaction data from almost all of the *C. elegans* literature published from 1993 to 2019. As of September 2019 (WormBase Release WS272), WormBase contains 32 669 physical protein–protein interactions for *C. elegans*. Within the dataset, 21 738 protein–protein interactions are unique, and 6678 unique genes are involved in these interactions. These interaction data were curated from 1492 peer-reviewed papers, which were selected from the literature by automatic text mining and manual verification. Since our 2018 update, the number of protein-protein interactions curated in WormBase has increased by 15.5%. All the physical interaction data in WormBase are supported by experimental evidence from original research papers. The curation details in WormBase and the statistical comparison with other databases for this data type are presented previously (5).

### Updates to WormBase ontologies

Over the years, WormBase has developed and maintained three ontologies that are regularly used for curation and data dissemination: the worm anatomy ontology (6), the worm phenotype ontology (7), and the worm development (life stage) ontology. All three ontologies are now available on GitHub: the anatomy ontology (https://github.com/obophenotype/c-elegans-gross-anatomy-ontology), the phenotype ontology (https://github.com/obophenotype/c-elegans-phenotype-ontology) and the development ontology (https://github.com/obophenotype/c-elegans-development-ontology). The release cycles for all three ontologies are managed using the Ontology Development Kit (ODK) (https://github.com/INCATools/ontology-development-kit). For every release, the ODK ensures that the ontology is using the most up-to-date versions of imported ontologies, and adheres to the quality control recommendations of the Open Biological and Biomedical Ontology (OBO) Foundry (http://obofoundry.org). Since 2018 WormBase curators have been active contributors to the Phenotype Reconciliation Effort (8) which aims at harmonizing the representation of phenotype terms across all phenotype ontologies. The reconciliation effort uses a templating approach to define patterns for the representation of phenotype terms. Implementing these templates has resulted in a more automated, complete and consistent classification of our phenotype terms and has begun the process of aligning our phenotype ontology with analogous ontologies from other organisms such as the Human Phenotype Ontology (HPO; (9)) or the Mammalian Phenotype Ontology (MP; (10)). Furthermore, the resulting richer axiomatization with links to other important ontologies such as the Gene Ontology (11) will make browsing of the ontology more complete and intuitive to end-users.

### Gene summaries: improved readability, more data and increased coverage

**Figure 1.**
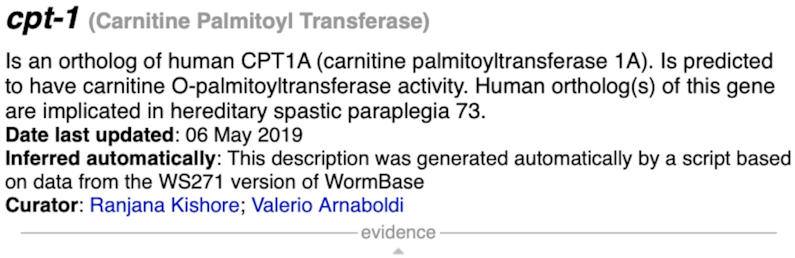
The gene description of *cpn-1* is an example of a description informed by the function of the orthologous human gene and the predicted protein domain signatures.

WormBase continues to implement and improve existing automation in the curation process in order to increase efficiency and to keep abreast of the sheer volume of published literature. Recently the gene summaries pipeline has been completely rewritten in order to improve data content and readability of the summaries, and to increase coverage of genes. Gene summaries are short text descriptions of gene function that are displayed in the ‘Overview widget’ on gene pages (e.g. Figure 1). These are automatically generated during each build of WormBase, based on curated and highly structured gene data that already exist in the database (12). New data content in the summaries includes the addition of cross-species data such as human ortholog disease implications in order to help in the discoverability of potential *C. elegans* models of human disease; this data is drawn from the Alliance of Genome Resources (https://www.alliancegenome.org; The Alliance of Genome Resources Consortium, accepted; NAR-02425-Data-E-2019.R1). Also of significance is over 15 000 information poor, lesser studied *C. elegans* genes that now have descriptions based on data related to protein domains, large scale expression and/or orthologous human gene molecular function, thus providing further clues about gene function. In total, as of the WS273 release of WormBase, over 160 000 gene summaries are available for ten nematode species.

## USER INTERFACE

### Graph Summary of Ontology-based Annotations

To help users comprehend the biological meaning that can be gleaned from large amounts of gene function annotations that we have curated, we present SObA (Summary of Ontology-based Annotations) graphs. We have expanded the coverage from phenotype ontology to four more ontologies: anatomy, disease, life stage and the gene ontology. On each gene page, one can find SObA graphs in the corresponding information widgets, including expression, gene ontology, human diseases and phenotypes. We also added a new feature in the graphs that allows users to interactively specify the level of hierarchy depth to display. This feature should help users to get the appropriate high-level summary view they want to see.

### Sequence widget improvements

Sequence widgets (found on the Variation, CDS, Transcript and Gene Pages) are some of the most heavily used components of the WormBase site, playing a central role in experimental design and verification. We have upgraded the generation of these widgets using modern technology to make them faster, more robust, and more programmatically useful. One of the major changes is that the REST Application Programming Interface (API; discussed below) that underpins these widgets now delivers native sequence that is formatted on the client's browser via the React JavaScript library. This small but subtle behind-the-scenes change results in a significantly improved experience for the user.

During this re-implementation, we took the opportunity to add several new features to the display. These enhancements include providing better markup that makes finding sequences much easier visually and native positive and negative strand representations for features like variations. The upshot of these improvements make it much easier to confirm reported variations with the scientific literature. At last, we made the option of downloading FASTA formatted sequence more prominent.

### Genome browser improvements

The JBrowse genome browser at WormBase has gained new data tracks and considerable functionality. There is a new section of tracks named ‘Externally sourced resources’ for displaying data hosted at other locations but may be useful to WormBase users. Among these resources include the nematode conservation track provided with the UCSC genome browser (13), PhyloCSF conservation tracks provided by the Broad Institute (https://data.broadinstitute.org/compbio1/PhyloCSFtracks/trackHub/hub.DOC.html), a track providing predicted sgRNA regions for use with CRISPR experiments (http://genome.sfu.ca/crispr/) and a track providing variation data for a variety of *C. elegans* strains (14). At last, an update to the underlying JBrowse software has made it possible for users to load a variety of binary data files like tabix or csi indexed files, as well as CRAM formatted files (15).

Several tracks that already existed have improved functionality, often reproducing functionality that already existed in the older GBrowse instance at WormBase. For example, the ‘EST (best)’ track now has a dashed line linking 5′ and 3′ reads making it easier to see the extent of an EST clone. There is a track that shows the frame of translation for protein coding transcripts reminiscent of the ‘cds’ glyph from GBrowse. In this case, it uses shades of blue that match up with identical shading in reference sequence (DNA) track to show the frame of translation. The RNASeq introns track got a considerable improvement where the data are split between two tracks, one that shows common predicted introns and one that shows more rare ones. The display of data from modENCODE (16) also is improved in JBrowse, where two improvements were made: the first was to create a separate JBrowse instance for modENCODE data that were mapped to an older assembly; the second addition was to display peak calls where available as pink highlights over the blue BigWig data using a plugin created by the JBrowse development team specifically for this purpose (https://github.com/cmdcolin/wigglehighlighter).

### WormMine data mining platform

WormMine is WormBase's most complete data integration service, based on the InterMine data warehousing framework (17). With a powerful and streamlined data integration and mining platform, WormMine offers a user-friendly interface with a large number of options for building complex and replicable queries. Since our last update, we have increased the number of data sources integrated into WormMine (Disease, Anatomy and Phenotype Ontology, Orthologs/Homologs and expanded protein sequences for additional species) and expanded our current data sources with additional fields. For example, we increased information available on Variations to include the effect on protein, and greatly improved relationships between many classes of information.

We continue to update WormMine in concert with improvements provided by the core Intermine team. Recently, we transitioned to Intermine 2.x, which has resulted in faster and more reliable querying, as well as improved build times. WormMine is hosted entirely on Amazon Web Services which we have leveraged to build a stable and reproducible build/deployment system.

### SimpleMine

SimpleMine is a data mining facility that dispenses with many of the complexities of other platforms in the interest of speed and simplicity. Users can enter a list of IDs, select checkboxes of the types of data they wish to retrieve and quickly retrieve that data. More fields were added to SimpleMine per users’ request. These include reference Uniprot ID, genetic map position, sequenced alleles and gene interactions. We also created a field called ‘Expression Cluster Summary’. For each gene in WormBase, Expression Cluster Summary gives a brief description on regulation and anatomical enrichment results derived from high-throughput experiments studies like microarray, RNA-seq and proteomics.

### Tools for querying expression data

SPELL (Serial Pattern of Expression Levels Locator) is a query-driven search engine for microarray, RNAseq and Proteomics data (18). WormBase maintains an independent instance of SPELL (https://spell.wormbase.org) populated with *C. elegans* data. Given a small set of query genes, SPELL identifies which datasets are most informative for these genes, then within those datasets, additional genes are identified with expression profiles most similar to the query set. WormBase SPELL has collected over 6000 experiments for nine nematode species. Users can also download these datasets for their own analysis. We have improved the performance of the WormBase SPELL instance over 2-fold since our last update.

Two RNA-seq related tools were added to WormBase. Users can now use ‘Expression Dataset Locator’ to browse and download processed data from ∼7000 genomic expression analyses published in ∼400 nematode research articles. The search may combine specific platforms, species, tissues and research topics. ‘RNAseq FPKM Gene Search’ allows users to enter or upload a list of genes from one of the selected species to get an HTML or Excel file with their FPKM (Fragments Per Kilobase of transcript per Million mapped reads) values in all RNAseq experiments that WormBase annotated. Users can set up filters to refine the results according to Strain, Life stage, tissue specificity or treatment.

### Programmatic REST API

**Figure 2.**
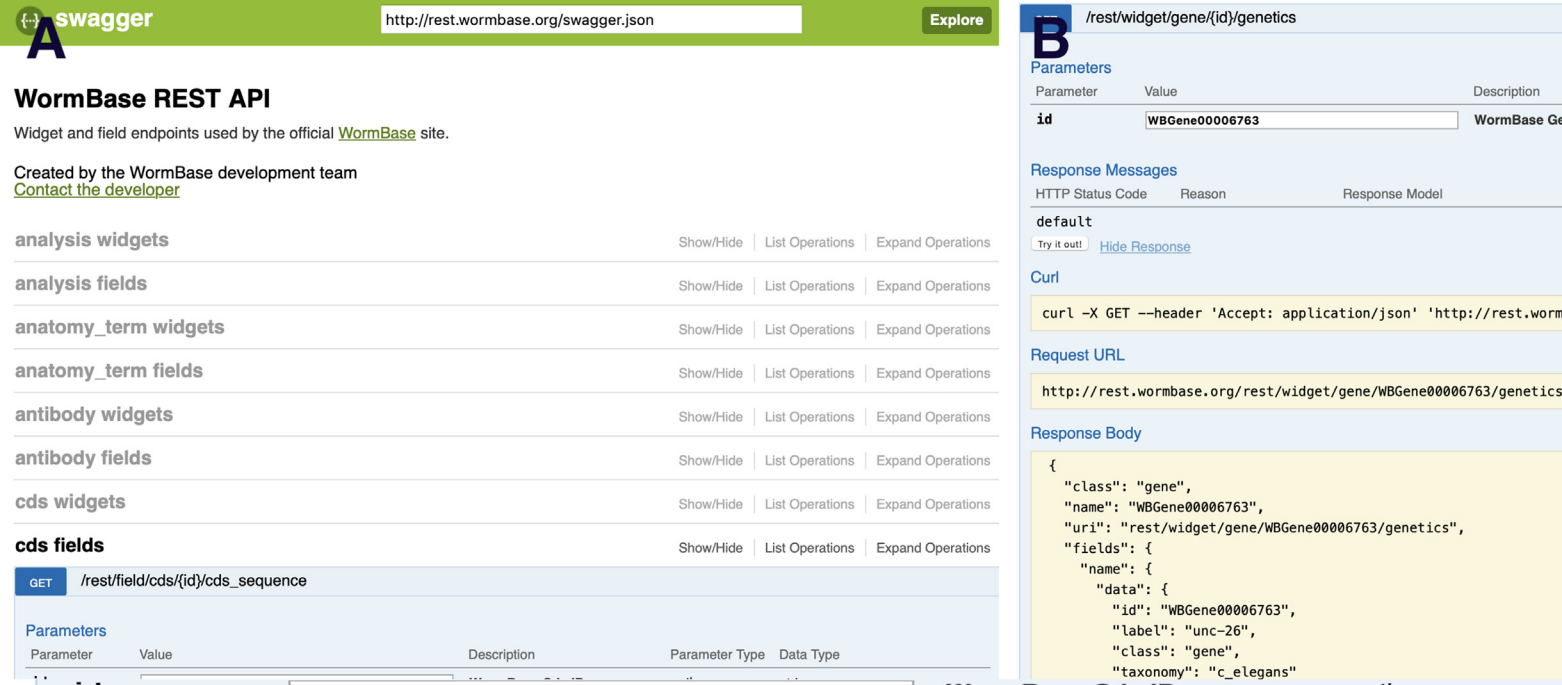
The Swagger (https://swagger.io) REST API interface at WormBase (http://rest.wormbase.org) makes it trivial to identify and test REST endpoints for creating programmatic data mining workflows. (**A**) A list of all endpoints. (**B**) A typical response.

The WormBase website is built on top of a RESTful API and we make this same API available for users interested in interacting with the resource programmatically. The documentation site (http://rest.wormbase.org/index.html) lists the available endpoints and parameters, and allows users to interact with the API through a web interface to explore the capabilities of the API without writing any code (Figure 2). This REST API is a flexible way of retrieving data from WormBase, but it comes with a steeper learning curve than a typical graphical user interface. Users intending to fetch data for many entities of the same type—and to do so periodically over time—should consider the REST API, particularly if WormMine or the downloadable files on the FTP site do not meet their needs.

## ARCHITECTURE

As a multi-decade project with electronic roots predating the web, WormBase must support deeply ingrained legacy systems while seamlessly transitioning to more modern technologies. We have extensively leveraged Amazon Web Services for over a decade to make major transitions behind the scenes with minimal impact to end users. To improve availability and reduce downtime of the WormBase website, we employ technologies like AWS Elastic Load Balancer, CloudWatch and Auto Scaling to monitor the health and resource utilization of our web servers, and automatically take remediating actions, such as replacing a faulty server when a fault is detected, or deploying more or less servers based on the traffic load to our servers.

We have also modernized the technologies upon which the website relies. As part of a multi-year effort to upgrade the software driving the website, we completed a major database migration from AceDB (19) to the cloud-native database management system Datomic (https://datomic.com/). This migration has resulted in major performance enhancements and increased the overall flexibility of our software stack. Leveraging technologies such as React, npm and webpack, we have built more responsive and feature-rich user interfaces and visualizations for sequences, ontology and interaction graphs and ontology ribbons. Along with new technologies, we maintain a diverse set of software built with technologies that lost popularity yet remain important to maintain for reproducibility. By turning these components into containers (self-contained packages of software and dependencies) and automating the steps of deployment, we can support these resources with reduced effort and specialized knowledge of those legacy technologies.

## USER SUPPORT

We offer users multiple options for assistance. Users can receive help in real-time directly from WormBase curators and developers (pending availability) via an in-line chat function directly on the website. Alternatively, our help desk fields queries at help@wormbase.org and on Twitter (https://twitter.com/wormbase; @wormbase). WormBase is also closely aligned with the rapid publishing platform microPublication (https://micropublication.org/; (20)) where authors can submit an article, receive a citable reference and have their data deposited directly in WormBase.

## FUTURE DIRECTIONS

As one of the founding members of the Alliance of Genome Resources project, WormBase is playing an active role in the development of this important integrative effort. We will continue to more closely align WormBase data, ontologies and curation processes with those of the Alliance. The Alliance greatly increases the amount of software development effort available to the *C. elegans* community. The Alliance portal (https://www.alliancegenome.org) has a uniform look and feel for human, *C. elegans* and select other model organism genes and key data; this site is now arguably the best way to find key information about the *C. elegans* ortholog of a human or fly gene.
